# The association between child *Schistosoma* spp. infections and morbidity in an irrigated rice region in Mali: A localized study

**DOI:** 10.1016/j.actatropica.2019.105115

**Published:** 2019-11

**Authors:** Ngoy Mutombo, Aly Landouré, Wing Young Man, Alan Fenwick, Robert Dembélé, Moussa Sacko, Adama D. Keita, Mamadou S. Traoré, Joanne P. Webster, Mary-Louise McLaws

**Affiliations:** a Epidemiology and Hospital Infection Prevention and Control, School of Public Health and Community Medicine, UNSW Medicine, UNSW Sydney, Australia; b Centre for Biomedical Research, Burnet Institute, Australia; c Institut National de Recherche en Santé Publique (INRSP), National Schistosomiasis Control Program, Bamako, Mali; d Schistosomiasis Control Initiative, Department of Infectious Disease Epidemiology, Imperial College London, London, United Kingdom; e Programme National de Lutte Contre la Schistosomiase, Ministère de la Santé, Bamako, Mali; f Service de la Radiologie, Hôpital National du Point G, Bamako, Mali; g Centre for Emerging, Endemic and Exotic Diseases, Department of Pathobiology and Population Sciences, Royal Veterinary College, University of London, AL9 7TA, UK

**Keywords:** Schistosomiasis, Human morbidity, Anaemia, Bladder, Micro-haematuria, Rice irrigated scheme, Mali

## Abstract

**Background:**

Schistosomiasis is one of the neglected tropical diseases endemic to Mali. There has been insufficient investigation of the morbidity burden in highly endemic irrigated rice areas with the ongoing mass drug administration with praziquantel. In February 2005, a year after an initial mass drug administration in 2004, we performed the first cross-sectional survey of schistosomiasis in the Kokry-Bozo village in the Office du Niger rice irrigation region. In the fourteen years since this survey, there has been almost no research into schistosomiasis morbidity in Mali due to lack of funding. Therefore, the 2005 survey supplies near-baseline data for any future research into the treatment impacts in the area.

**Methods:**

One hundred and ninety-four children aged 6–14 years from two schools were assessed for bladder pathology by ultrasound, and for anaemia and micro-haematuria by laboratory tests. *Schistosoma* eggs were examined microscopically in fresh stool and urine samples. Multivariate logistic regression analysis quantified the association of *Schistosoma* infections with anaemia, bladder pathology and micro-haematuria. Akaike’s information criterion was used to test the assumption of linear effects of infection intensity classes and used to compare across models.

**Results:**

The overall prevalence of schistosomiasis in 189 school children was 97%; 17% (33/189) had a single infection (*S. mansoni,*13%, or *S. haematobium,* 4%) and 80% (156/189) were co-infected with *S. mansoni and S. haematobium*. The overall prevalence of *S. mansoni* with light infection was 27% (53/194), moderate infection was 24% (47/194) and heavy infection was 42% (81/194). Of the 194 of children investigated for *S. haematobium* 59% (114/194) had light infection and 26% (50/194) had heavy infection. No hookworm eggs were detected.

The level of abnormal bladder pathology was 18% (35/189) with the highest found in 10–14 year old children. The prevalence of anaemia was 91% (172/189) and was twice as likely to be associated (OR 2.0, 95% CI 1.1–3.9) with *S. mansoni* infections than in children without infection. As infection intensity with *S. mansoni* increased the risk of anaemia (OR 2.0, 95% CI 1.1–3.9) also increased. As infection intensity with *S. haematobium* increased bladder pathology (OR 2.4, 95%CI 1.3–4.5), haematuria (OR 6.7, 95%CI 3.3–13.6) and micro-haematuria increased (OR 2.4, 95%CI 1.3–4.5).

**Conclusion:**

Our research contributes an important micro-geographical assessment of the heavy burden of schistosomiasis and associated morbidity in children who live in the rice irrigation regions. Our literature review found that there has been very limited research conducted on the impact of the treatment to control morbidity in the ON. Therefore, there is a need to do a comparable, but more extensive, study to identify any changes in morbidity and to indicate current requirements for the control programme. Our results from 2005 called for routine integration of iron supplementation, food fortification and diet diversification into the deworming program.

## 1 Introduction

Schistosomiasis is a chronic and debilitating disease that affects 600 million people in developing countries of whom 85% are located in sub-Saharan Africa and an estimated 250 million worldwide require treatment (Colley et al., 2017). An estimated 20 million infected persons currently suffer from a severe form of the disease (Colley et al., 2017) resulting in more than 300,000 related deaths annually (King and Dangerfield-Cha, 2008; Southgate et al., 2005; van der Werf et al., 2003).

Four major forms of human schistosomiasis (*S. mansoni*, *S. haematobium*, *S. intercalatum* and *S. guineensis*) are recognised in sub-Saharan Africa. The different clinical manifestations are related to the species-specific egg-laying sites: the mesenteric venous systems for intestinal *Schistosoma mansoni*, *S. intercalatum* and *S. guineensis*, as well as the vesical veins of the urogenital system for *S. haematobium* (Barsoum et al., 2013; Vennervald and Dunne, 2004) and *Schistosoma* spp. hybrids (e.g. *S. haematobium*/*S. curassoni* and *S. haematobium*/*S. bovis* hybrids) (King et al., 2015; Leger and Webster, 2017).

*Schistosoma* spp. eggs cause most of the pathology ranging from anaemia, haematuria, bloody diarrhoea and abdominal pain, to organ-specific effects such as chronic hepatosplenism, periportal fibrosis, and ureteral and/or bladder fibrosis, calcification of urinary tract and bladder cancer (Colley et al., 2014). The mainstay of the current control strategy against schistosomiasis in endemic regions consists of preventive chemotherapy (PCT) with mass drug administration (MDA) with praziquantel (PZQ), to reduce infection and associated morbidity through regular treatment (WHO, 2012). Longitudinal cohort follow-up morbidity data are essential for long term impact monitoring and evaluation of PCT strategy. However, control programmes typically collect prevalence and intensity of infection data and rarely morbidity data, due to its high cost.

In Mali, both urogenital and intestinal forms of schistosomiasis remain endemic throughout the country with geographically varying degrees of prevalence (Clements et al., 2008; Dabo et al., 2015) yet clinical and population-based data are scarce because reports focus on prevalence and infection intensity rather than associated morbidities (Personal communication with Moussa Sacko, National Schistosomiasis Control Program, Mali). Since the start of the 2004 schistosomiasis control program in Mali, baseline and follow-up surveys showed that some areas are reducing well, others have ongoing very high infection prevalence and intensities for both *S. mansoni* and *S. haematobium* infections. One of the highest ongoing endemicity areas is that of the Office du Niger (ON), Ségou region, despite control with regular, annual PCT (Landoure et al., 2012). School age children in this area are almost universally infected with *Schistosoma* spp**.** The Office du Niger is a rice-irrigation area which harbors two main *Schistosoma* species, *S. haematobium* and *S. mansoni*. A control effort with PZQ drug and mollusciciding programs in Mali commenced in the early 1970s, then again between 1982 and 1992, focusing on the ON and the Plateau Dogon region with funds from the German Technical Co-operation (GTZ) (Brinkmann et al., 1988; Traore et al., 1998). A new initiative of national schistosomiasis control program of MDA commenced in 2004 under the Schistosomiasis Control Initiative (SCI) (Clements et al., 2009; SCI) that targeted school children with a single dose (40 mg/kg) of PZQ. Since 2007, MDA with PZQ has been steadily scaled up achieving 100% geographical coverage and 72–100% programme coverage as part of the integrated national control programme on NTDs, funded by the United States Agency for International Development (USAID) (Dembele et al., 2012). Throughout the ongoing control program morbidity data (anaemia, haematuria, bladder pathology, liver and spleen morbidity) have not been routinely collected due to lack of resources. The aim of our study was to assess schistosome-induced morbidity in *S. haematobium* and *S. mansoni* infections with a particular hypothesis that the estimation of schistosome infections-related morbidity at micro-geographical level might be an indicator of the degree of the pathologies in an endemic irrigated region. Here we reported the morbidity associated with schistosomiasis that include anaemia, bladder pathology and haematuria, as a micro-epidemiological evaluation of the initial success of the PZQ chemotherapy program in the region.

## 2 Materials and methods

### 2.1 Study area, population and design

At the start of the SCI MDA program for Mali, a random selection of 194 school-age children aged 6–14 years were surveyed from Kokry-Bozo village in the Office du Niger rice irrigation scheme of Mali. The cross-sectional study was completed during the 2005 SCI survey (described in full (Koukounari et al., 2010)). Previous parasitological and morbidity baseline surveys in the ON occurred during March and April of 2004 while the follow-up survey was performed one year later in May 2005. Since 2004, Ségou and Macina districts (located in Ségou region) received regular yearly rounds of MDA till 2018 except in 2007. At the time of our study, school children in this area had received two MDA treatments with PZQ: (a) one after baseline data collection in March-April 2004 and (b) another during preventive chemotherapy intervention in February 2005. Kokry-Bozo baseline morbidity data on anaemia, haematuria and abnormal bladder/liver/spleen pathologies were only collected for the February 2005 survey. We report findings from the MDA control programme that have not been previously published by (Koukounari et al., 2010). The Office du Niger is known *a priori* to be highly endemic for schistosomiasis and malaria (Clements et al., 2009; Sogoba et al., 2007; Stecher et al., 2017). Children provided fresh urine, stool and finger-prick blood samples and had ultrasonographic assessment for bladder abnormality.

### 2.2 Parasitological examinations

Stool and urine samples were collected from children between 09:00 and 13:00 h. Duplicate urine syringe filtration slides (Peters et al., 1976) were used to determine *S. haematobium* prevalence and intensity. Duplicate Kato-Katz (K-K) thick smear slides (Katz et al., 1972) were used to define the prevalence and intensity of *S. mansoni* infection. Duplicate testing on the same stool or urine sample was performed once for each child due to logistical constraints although replicate stool samples over several days improves the accuracy of estimates of the intensity of schistosomiasis (de Vlas et al., 1993; Teesdale et al., 1985). The mean number of eggs per 10 ml of urine for *S. haematobium* and the mean number of eggs per gram (EPG) of stool for *S. mansoni* were used to define intensity of infections as to low, moderate or high intensity in accordance with World Health Organization established intensity cut-off values (WHO, 2006). All laboratory tests were performed by the same experienced laboratory technicians from the Malian National Public Health Research Institute.

### 2.3 Determination of haemoglobin concentrations

Haemoglobin (Hb) was estimated from 100*μ*l of finger prick blood using a portable battery-operated HemoCue® photometer (HemoCue, Angelholm, Sweden) (von Schenck et al., 1986). The same experienced clinical nurses performed all blood collection who were blinded to parasitological results. Anaemia was defined for age and sex in accordance with the WHO guidelines (WHO, 2001). Anaemia was defined as an Hb<11.5 g/dl for children aged 6–11 years, Hb<12.0 g/dl for children aged 12–13 years, Hb<13.0 g/dl for males Hb>13 years and Hb<12.0 g/dl for girls Hb>13 years (WHO, 2011). Severity of anaemia was classified (WHO, 2011) as: severe Hb<7.0 g/dl, moderate Hb 7.0–9.9 g/dl, mild Hb10–11.9 for girls and boys ≤12 years, and boys >13 years as Hb 10–12.99 g/dl (Table 1).

**Table 1:** Prevalence of anaemia among 189 children by age and sex.

Demographics	Anaemia status % [95%%CI] (N)				OR [95% CI]	P (χ2)
	Severe	Moderate	Mild	Normal		
*Sex*						
Male (n = 88)	8 [3-15] (7)	55 [44-65] (48)	34 [25-44] (30)	3 [1-9] (3)	4.5 [1.3-20.3]	0.01 (6.28)
Female (n = 101)	5 [2-11] (5)	40 [30-49] (40)	42 [33-51] (42)	14 [8-22] (14)	1	
*Age*						
6-9 years (n = 67)	7 [3-16] (5)	37[26-49] (25)	40 [29-52] (27)	15 [8-25] (10)	1	
10-14 years (n = 122)	6 [5-11] (7)	52 [43-61] (63)	37 [29-46] (45)	6 [2-11] (7)	2.9 [1.04-8.4]	0.03 (4.46)
**Total (N = 189)**	**6 [3-10] (12)**	**47 [39-54] (88)**	**38 [31-5] (72)**	**9 [5-14] (17)**		

P (χ^2^): *P*-value and χ^2^ from Pearson chi-square test of normal status versus all other anaemic status.

### 2.4 Urine examination for micro-haematuria

Urine reagent strips (Hemastix®; Bayer, Tarrytown, NY) were used to detect blood in urine specimens (French et al., 2007). The level of micro-haematuria (invisible haematuria) was graded semi-quantitatively as negative, trace haemolysed or trace non-haemolysed. Non-haemolysed was graded in accordance with the manufacturer recommendations as: + (˜25 erythrocytes/*μ*l); ++ (˜80 erythrocytes/*μ*l); +++ (˜200 erythrocytes/*μ*l).

### 2.5 Ultrasound examination

A portable ultrasonography device (SSD-500®; Aloka, Tokyo, Japan) equipped with a convex 3.5-Mhz transducer (probe) was used for bladder examination (described in detail (Gouvras et al., 2013; Koukounari et al., 2010; Sacko et al., 2011) in children with abnormal pathology and performed by the same physician trained in the Niamey WHO protocol (Richter et al., 2001, 2000; WHO, 2000).

### 2.6 Statistical analyses

Data management and statistical analysis were performed using SPSS 17.0 (SPSS Inc., Chicago, IL, USA) and Stata v.11.0 (Stata Corp., 2011) with chi-square (χ2) test for linear trend performed by Stata v.11.0 (Stata Corp., 2011). Pearson’s chi-square test was used to test significance in differences between normal Hb level compared with three levels of anaemia and bladder pathology and haematuria by age and sex. The percentage of children displaying anaemia, bladder pathology and haematuria were determined by infection status: single species infections, co-infected or uninfected.

Multivariate logistic regression models were fitted using Stata v.11.0 (Stata Corp., 2011) to enable the associations between several covariates on the pathological manifestations to be tested simultaneously. Odds Ratios (OR), 95% Confidence Intervals (95%CI), *P*-values and z test were established from multivariate logistic regression analysis for urogenital-related morbidities, haematuria and anaemia pathologies. Separate models were developed for each of the morbidity outcomes of abnormal bladder pathology, micro-haematuria and anaemia structured as binary variables. Potential predictors included in the model were infection intensity (classified on infection intensities category using WHO standards), age and sex. The assumption of linear effects of infection intensity classes were tested using Akaike’s Information Criterion (AIC) (Vandekerckhove et al., 2014) to compare models fitted for separate effects for each combination of *S. mansoni* and *S. haematobium* intensity classes. The AIC comparisons identified from the models fitting sex, age group and infection intensity classes) yielded better fit. When linear effects were incorporated, the intensity classes for *S. haematobium* infection were assigned 0 for ‘not infected’ (0 egg/10 ml), 1 for ‘light’ (1–49 eggs/10 ml) and 2 for ‘heavy’ (≥ 50 eggs/10 ml). Intensity classes for *S. mansoni* infection were assigned 0 for ‘not infected’ (0 epg), 1 for ‘light’ (1–99 epg), 2 for ‘medium’ (100–399 epg) and 3 for ‘heavy’ (≥ 400 epg). Children were categorised into two age groups: younger age 6–9 years and older age 10–14 years. *P*-values of ≤0.05, using a two-sided test, were considered statistically significant. The sample size does not provide precision to less than one decimal places therefore odd ratios were rounded up to one decimal place for all odds >0.99. Proportions were also rounded up.

### 2.7 Ethical considerations

Ethics approval was obtained from the Mali Ministry of Health, London Research Ethics Committee of the Imperial College (EC NO: 03.36. R&D No: 03/SB/033E) and the University of New South Wales (UNSW Sydney). Prior to the recruitment of the children, verbal informed consent was first obtained from the parents/guardians and then each child was asked for his/her verbal consent as participation was voluntary. Consent was recorded with the village committee that comprised of parents, teacher's director, teachers and community leader. All children found to be diagnosed with schistosomiasis were treated in accordance with the WHO recommended dose of PZQ (40 mg/kg).

## 3 Results

A total of 194 schoolchildren aged 6–14 years were enrolled. The mean age was 10.5 years and the ratio of girls to boys was 1.1:1. Nearly all (97%; 189/194) participants tested positive for at least one schistosome species; 80% (155/194) co-infection with *S. haematobium/S. mansoni* and 17% (33/194) single parasitic infection (13% *S. mansoni*; 4% *S. haematobium*). The prevalence of *S. mansoni* by intensity was 42% (81/194) heavy, 24% (47/194) moderate and 27% (53/194) light. The prevalence of *S. haematobium* by intensity was 26% (50/194) for heavy and 59% (114/194) for light.

### 3.1 Prevalence of pathological manifestations

#### 3.1.1 Anaemia

Anaemia was diagnosed in 91% (172/188) of children infected regardless of schistosome species (Table 1). The mean Hb level for all children was 10.5 g/dl (95%CI 9.8–11.2 g/dl). The prevalence of severe, moderate and mild anaemia was 6%, 47% and 38% respectively. Although the prevalence of anaemia was high in both males and females infected (96% and 86% respectively) and was 4.5 times more likely to be diagnosed in males than females (OR 4.5, 95%CI 1.3–20.3, *P = 0.01*) (Table 1). The prevalence of anaemia was high in all infected children and anaemia was 2.9 times more likely to be diagnosed in older children than younger children (OR 2.9, 95%CI 1.04–8.0, *P = 0.03*).

#### 3.1.2 Bladder pathology and micro-haematuria

Bladder pathology was present in 18% of children and did not significantly differ by sex (OR 1.01, 95%CI 0.5–2.1, *P = 0.87*) or age (OR 0.84, 95%CI 0.37–1.8, *P* = 0.67) (Table 2). Micro-haematuria was present in 60% of children and did not significantly differ by sex (OR = 1.3, 95%CI 0.73–2.3, *P = 0.36*) or age (OR 0.71, 95%CI 0.38–1.3, *P = 0.27*).

**Table 2:** Frequency of bladder pathology and micro-haematuria among 194 children.

Demographics	Bladder pathology			Micro-haematuria		
	%[95%CI] (N)	OR [95%CI]	P (χ2)	% [95%CI] (N)	OR [95%CI]	P (χ2)
**Sex**						
Male (n = 93)	19 [11-27] (17)	1		59 [47-67] (53)	1	
Female (n = 101)	18 [11-26] (18)	1.01 [0.5-2.1]	0.87 (0.03)	63 [54-72] (64)	1.3 [0.73-2.3]	0.36 (0.82)
**Age**						
6-9 years (n = 67)	16 [9-27] (11)	1		66 [54-76] (44)	1	
10-14years (n = 127)	19 [13-26] (24)	0.84 [0.37-1.8]	0.67 (0.18)	59 [49-66] (73)	0.71 [0.38-1.3]	0.27 (1.23)
**Total**	**18 [13-24] (35)**			**60 [53-67] (117)**		

#### 3.1.3 Bladder pathology, haematuria and anaemia by infection

The prevalence of bladder pathology in children with a co-infection of *S. haematobium* and *S. mansoni* was 19% (95%CI 14%–26%, 29/156) (not shown in Tables). More than half (61%, 95%CI 54%–68%, 116/189) the infected children had haematuria with anaemia present in almost all infected children: *S. haematobium* only (87%, 95%CI 18%–92%, 7/8), *S. mansoni* only (95%, 95%CI 71%–97%, 24/25), co-infected (90%, 95%CI 59%–74%, 140/156).

### 3.2 Logistic regression analyses for the association between the intensity of schistosomiasis infection status, gender, age and pathological manifestations

As infection intensity of *S. haematobium* increased bladder pathology (OR 2.4, 95%CI 1.3-4.5*, P*< 0.01) and haematuria (OR 6.7, 95%CI 3.3-13.6*, P*< 0.001) were significantly more common (Table 3). As infection intensity increased with *S. mansoni* anaemia (OR 2.0, 95% CI 1.1-3.9*, P*< 0.05) was significantly more common.

**Table 3:** Linear covariate analysis for risk factors associated with pathology in 194 children.

	Bladder pathology		Haematuria		Anaemia	
	OR (95%CI)	P (z)	OR (95%CI)	P (z)	OR (95%CI)	P (z)
Male	1		1		1	
Female	0.96 (0.44-2.1)	0.92 (-0.10)	1.43 (0.73- 2.8)	0.99 (1.06)	0.32 (0.08-1.3)	0.11 (-1.61)
6-9 years	1		1		1	
10-14 years	1.5 (0.65- 3.4)	0.34 (0.95)	1.02 (0.50-2.1)	0.94 (0.07)	1.9 (0.58- 6.4)	0.28 (1.08)
Infection intensity						
*S. mansoni*	0.83 (0.55-1.2)	0.36 (-0.90)	0.99 (0.69-1.4)	0.96 (-0.04)	2.0 (1.1-3.9)	0.03 (2.17)
*S. haematobium*	2.4 (1.3- 4.5)	0.007 (2.68)	6.7 (3.29-13.60)	<0.001 (5.26)	0.55 (0.21- 1.4)	0.22 (-1.21)

## 4 Discussion

A regular morbidity data collection has not been conducted countrywide in Mali for long term impact monitoring of MDA since our 2005 survey due to lack of resources. Records from the National Schistosomiasis Control Program and results from the literature review show that since our February 2005 survey, only very limited morbidity data (anaemia, haematuria and organomely) were collected in three villages from the Ségou district. These were located just outside the Office du Niger. This occurred in 2007–2009 as part of a larger multidisciplinary study (Stecher et al., 2017). The second study was by Sacko et al. (2011) on urinary tract pathology, nutritional status and anaemia in Koulikoro and Selingue, in 6 villages, far away from the Office du Niger. From the 2005 research we have quite a unique dataset on anaemia, haematuria and bladder pathology one year from the start of the MDA program (near-baseline data) concerning schistosomiasis in children in the Office du Niger, Macina district, where there is an ongoing MDA with PZQ in Mali. This is key data to compare with future surveys to enable the effectiveness of the ongoing MDA program to be evaluated.

Key findings of this micro-epidemiological study were the strikingly high prevalence of pathologies that included anaemia in 91% of surveyed children and that 80% of the children examined were co-infected with *S. mansoni* and *S. haematobium.* The morbidities that were identified were likely attributable to schistosomiasis, given the hyper-endemicity of this parasite in our children, although a component of the anaemia observed may be due to other factors. Our findings concur with other similar findings (Brinkmann et al., 1988; Clements et al., 2009; Koukounari et al., 2008; Landoure et al., 2012).

There is evidence that the length of exposure to schistosomiasis, and host and parasite genetics may impact a child’s response to PZQ as well as the child’s host response ability to modulate reactions to the parasite (Vennervald and Dunne, 2004). The likely cause of high intensity of infections and associated morbidity in our village could have been a result of an interruption to the mollusciciding programs and inappropriate MDA coverage prior to 2004. Co-infections of schistosomes only or in association with other parasites (e.g. STH and *P. falciparum*) in children elevates risk of morbidity (Ezeamama et al., 2008; Midzi et al., 2010; Pullan et al., 2011). Higher *S. haematobium*- and *S. mansoni*-associated morbidity in co-infections relative to single *Schistosoma* infections have been reported in Mali (Koukounari et al., 2010) and Kenya (Gouvras et al., 2013).

### 4.1 Anaemia

The results of our study show that anaemia was almost universal. The prevalence of anaemia in our study, 91%, was higher than the average 40% for Mali in school-age children, suggesting anaemia is a serious public health problem in the region. A recent study in three villages located just outside the ON support this (Stecher et al., 2017). In the region of the Office du Niger the rate of anaemia mirrors Mali with 84% in preschool-age children and 58% in women of reproductive age (SPRING, 2016).

Anaemia in our children was significantly associated with the intensity of *S. mansoni* infection, concurring with a report from Tanzania (Ajanga et al., 2006) while in contrast to Uganda (Tukahebwa et al., 2013). We believe our findings may indicate a unique relationship between *S. mansoni* infection and anaemia possibly in part anaemia of inflammation, the result of an inflammatory mechanism causing a high level of serum ferritin (>100 ng/mL) due to iron sequestration (storage) from iron-dependent host tissues (Butler et al., 2012; Foote et al., 2013).

The anaemia detected in children with *S. mansoni* infection may be related to insufficient iron intake, haemoglobinopathies, micronutrient deficiencies (vitamin A, B12, folate and riboflavin) (Midzi et al., 2010; Pullan et al., 2013; Soares Magalhaes and Clements, 2011; Thoradeniya et al., 2006) and social factors (Soares Magalhaes and Clements, 2011). Other risk factors include parasitic infections such as *Plasmodium falciparum* malaria (Pullan and Brooker, 2008; Pullan et al., 2013; Sogoba et al., 2007), *H. nana* (Oliveira et al., 2015), and hookworm infection (Fuseini et al., 2010; Koukounari et al., 2008, 2006; Magalhaes and Clements, 2011; Olsen et al., 1998; Pullan et al., 2011; Stoltzfus et al., 1997; Sturrock, 2001). We found the prevalence of *Hymenolepsis nana* to be very low in our study area and we could not detect STH in our 2007 investigation (unpublished).

Contrary to other reports from Ségou district (Stecher et al., 2017), Selengue and Koulikouro regions (Sacko et al., 2011; Stecher et al., 2017) we did not find an association between *S. haematobium* infection and anaemia (Table 3 and S2) and this may be due to small sample size.

We found anaemia was hyper-endemic in both females, 86%, and males, 96%, while other reports found anaemia to be more common in male children (Lwambo et al., 2000; Mupfasoni et al., 2009; Stecher et al., 2017). Our finding that anaemia was more common in 10–14 year olds than younger children, 6–9 years of age, concurred with others (Anumudu et al., 2008; Hall et al., 2001; Ullah et al., 2014) while others have reported anaemia actually improved in teenage years (Lwambo et al., 2000; Verma et al., 1998). Of note, a recent study in Ségou district shows that anemia related to *S. haematobium* infection was most pronounced in the 2–5 year olds males across ages and genders (Stecher et al., 2017), suggesting anaemia is a serious public health problem in under 5 years old children. We believe our finding reflects a greater exposure to contaminated water in our older children while they assist their families fishing and farm irrigation as well as during leisure time (Booth et al., 2004; Scott et al., 2003). Control of this exposure requires a culture change where children in the Kokry-Bozo region are kept away from working and playing in the water.

Multiple causes of anaemia present a challenge when designing effective intervention programs. Regardless of cause, iron supplementation in Kokry-Bozo needs to be considered for distribution as a therapeutic and preventive strategy. However, appropriate PZQ dosing and duration would eliminate an important risk factor, *S. mansoni*, for anaemia. With potential multiple risk factors for anaemia in the Kokry-Bozo region simultaneous control strategies for malaria (Sogoba et al., 2007) and addressing poverty (DNSI, 2007) are needed.

### 4.2 Bladder pathology and haematuria

Our findings suggest that bladder pathology and micro-haematuria were not infrequently diagnosed in children with *Schistosoma* infection from Kokry-Bozo village. According to a 2007–2009 study in three villages in Ségou district (located just outside the ON), 61% of individuals of both sexes across 2–40 years of age have micro-haematuria related to *S. haematobium* infection (Stecher et al., 2017). This is in line with our finding, suggesting that micro-haematuria continues to be a serious public health issue in the Ségou region. A 2010 study conducted in two different areas (Koulikoro district and Selingué dam) confirms our observation of high level of urinary pathology in Mali (Sacko et al., 2011).

Currently the prevalence of bladder pathology and haematuria in children not infected or infected with only one species of schistosomiasis was not estimated the small sample size. But a simple history of blood in children’s urine could provide a good indication of *S. haematobium* (Kapito-Tembo et al., 2009; Keiser et al., 2002; King and Bertsch, 2013; Lengeler et al., 2002; van der Werf et al., 2004). The exact mechanisms behind the micro-haematuria and bladder pathology in children infected with only *S. mansoni* may be the result of the low sensitivity of the urine filtration method failing to detect a mild urinary *S haematobium* infection (Knopp et al., 2015), bacteria urinary tract infection, glomerulonephritis, urinary tract tumour and sickle cell anaemia (Dawam et al., 2001; McDonald et al., 2006). Our findings of co-infections might suggest inter-species pairing has occurred where *S. haematobium* males paired with female *S. mansoni* that deviated the eggs to the urinary tract (Cunin et al., 2003; Koukounari et al., 2010). But even if this uncommon pairing had occurred it does not explain the prevalence of haematuria.

## 5 Limitations and strengths

The interpretation of the morbidity results is limited by a lack of adjustment for potential confounding factors that are not easy to measure or statistically control such as: degree and duration of exposure to schistosome pathogens, other parasitic diseases, viral infections, poor nutrition or micronutrient deficiencies, inherited haemoglobinopathies and thalassemia. In particular, poor sanitation, high levels of water contact and poor resources for decontamination of water observed during field trips could be major confounders. Research into improved statistical and epidemiological adjustment for these confounders in small village studies would be beneficial. Notwithstanding this methodological shortfall, our use of ultrasonography demonstrated a significant burden of abnormal bladder pathology in our sample of co-infected children. This is an important avenue for further research.

## 6 Conclusion

Aetiological factors including the degree that schistosomiasis contributes to anaemia, bladder pathology and haematuria may be difficult to determine, especially at the level of village surveys. Our results indicate that there was association between level of morbidity and infection intensity. Our study has provided a micro-epidemiological insight into morbidities in a village where the prevalence of schistosomiasis has remained hyper-endemic following one round of treatment. This treatment was part of the SCI MDA program and has continued from 2005 until now. Morbidities we measured were so commonplace in the school children that control strategies should include concurrent improvement in water and sanitation as well mollusciding, micronutrients supplementations and malaria control. Due to limited morbidity data between 2006 and 2019, there is a need for a comparable but more extensive study. It is necessary to identify any changes in morbidity and to indicate current requirements for the control programme in Mali.

## Authors’ contributions

The study conception and fieldwork were developed and performed by JPW and NM and analysis under the supervision of MLM and WYM. The manuscript was drafted by NM and MLM and all authors contributed to the critical revision of the manuscript for important intellectual content and agreed on submission.

## Funding

The Schistosomiasis Control initiative London, United Kingdom was supported by Bill and Melinda Gates Foundation and the EU Contrast Grants (CONTRAST/EUINCO. Dev contract no: 032203).

## Data profile

The research data are confidential. Survey respondents were assured the original data would remain confidential and will only be shared on request after consideration and delinking of any identification.

## Declaration of Competing Interest

The authors have declared that no competing interests exist.

## References

[bib0005] Ajanga A., Lwambo N.J., Blair L., Nyandindi U., Fenwick A., Brooker S. (2006). Schistosoma mansoni in pregnancy and associations with anaemia in northwest Tanzania. Trans. R. Soc. Trop. Med. Hyg..

[bib0010] Anumudu C., Afolami M., Igwe C., Nwagwu M., Keshinro O. (2008). Nutritional anaemia and malaria in preschool and school age children. Ann. Afr. Med..

[bib0015] Barsoum R.S., Esmat G., El-Baz T. (2013). Human schistosomiasis: clinical perspective: review. J. Adv. Res..

[bib0020] Booth M., Vennervald B.J., Kabatereine N.B., Kazibwe F., Ouma J.H., Kariuki C.H., Muchiri E., Kadzo H., Ireri E., Kimani G., Mwatha J.K., Dunne D.W. (2004). Hepatosplenic morbidity in two neighbouring communities in Uganda with high levels of Schistosoma mansoni infection but very different durations of residence. Trans. R. Soc. Trop. Med. Hyg..

[bib0025] Brinkmann U.K., Werler C., Traore M., Korte R. (1988). The national schistosomiasis control programme in Mali, objectives, organization, results. Trop. Med. Parasitol..

[bib0030] Butler S.E., Muok E.M., Montgomery S.P., Odhiambo K., Mwinzi P.M., Secor W.E., Karanja D.M. (2012). Mechanism of anemia in Schistosoma mansoni-infected school children in Western Kenya. Am. J. Trop. Med. Hyg..

[bib0035] Clements A.C., Bosque-Oliva E., Sacko M., Landoure A., Dembele R., Traore M., Coulibaly G., Gabrielli A.F., Fenwick A., Brooker S. (2009). A comparative study of the spatial distribution of schistosomiasis in Mali in 1984–1989 and 2004–2006. PLoS Negl. Trop. Dis..

[bib0040] Clements A.C., Garba A., Sacko M., Toure S., Dembele R., Landoure A., Bosque-Oliva E., Gabrielli A.F., Fenwick A. (2008). Mapping the probability of schistosomiasis and associated uncertainty, West Africa. Emerg. Infect. Dis..

[bib0045] Colley D.G., Andros T.S., Campbell C.H. (2017). Schistosomiasis is more prevalent than previously thought: what does it mean for public health goals, policies, strategies, guidelines and intervention programs?. Infect. Dis. Poverty.

[bib0050] Colley D.G., Bustinduy A.L., Secor W.E., King C.H. (2014). Human schistosomiasis. Lancet.

[bib0055] Cunin P., Tchuem Tchuente L.A., Poste B., Djibrilla K., Martin P.M. (2003). Interactions between Schistosoma haematobium and Schistosoma mansoni in humans in north Cameroon. Trop. Med. Int. Health: TM & IH.

[bib0060] Dabo A., Diarra A.Z., Machault V., Toure O., Niambele D.S., Kante A., Ongoiba A., Doumbo O. (2015). Urban schistosomiasis and associated determinant factors among school children in Bamako, Mali, West Africa. Infect. Dis. Poverty.

[bib0065] Dawam D., Kalayi G.D., Osuide J.A., Muhammad I., Garg S.K. (2001). Haematuria in Africa: is the pattern changing?. BJU Int..

[bib0070] de Vlas S.J., Gryseels B., van Oortmarssen G.J., Polderman A.M., Habbema J.D. (1993). A pocket chart to estimate true Schistosoma mansoni prevalences. Parasitol. Today.

[bib0075] Dembele M., Bamani S., Dembele R., Traore M.O., Goita S., Traore M.N., Sidibe A.K., Sam L., Tuinsma M., Toubali E., Macarthur C., Baker S.K., Zhang Y. (2012). Implementing preventive chemotherapy through an integrated National Neglected Tropical Disease Control Program in Mali. PLoS Negl.Trop. Dis..

[bib0080] DNSI (2007). Annuaire Statistique 2006.

[bib0085] Ezeamama A.E., McGarvey S.T., Acosta L.P., Zierler S., Manalo D.L., Wu H.W., Kurtis J.D., Mor V., Olveda R.M., Friedman J.F. (2008). The synergistic effect of concomitant schistosomiasis, hookworm, and trichuris infections on children’s anemia burden. PLoS Negl. Trop. Dis..

[bib0090] Foote E.M., Sullivan K.M., Ruth L.J., Oremo J., Sadumah I., Williams T.N., Suchdev P.S. (2013). Determinants of anemia among preschool children in rural, western Kenya. Am. J. Trop. Med. Hyg..

[bib0095] French M.D., Rollinson D., Basanez M.G., Mgeni A.F., Khamis I.S., Stothard J.R. (2007). School-based control of urinary schistosomiasis on Zanzibar, Tanzania: monitoring micro-haematuria with reagent strips as a rapid urological assessment. J. Pediatr. Urol..

[bib0100] Fuseini G., Edoh D., Kalifa B.G., Hamid A.-B., Knight D. (2010). Parasitic infections and anaemia during pregnancy in the Kassena-Nankana district of Northern Ghana. JHPE.

[bib0105] Gouvras A.N., Kariuki C., Koukounari A., Norton A.J., Lange C.N., Ireri E., Fenwick A., Mkoji G.M., Webster J.P. (2013). The impact of single versus mixed Schistosoma haematobium and S. mansoni infections on morbidity profiles amongst school-children in Taveta, Kenya. Acta Tropica.

[bib0110] Hall A., Bobrow E., Brooker S., Jukes M., Nokes K., Lambo J., Guyatt H., Bundy D., Adjei S., Wen S.T., Satoto Subagio H., Rafiluddin M.Z., Miguel T., Moulin S., de Graft Johnson J., Mukaka M., Roschnik N., Sacko M., Zacher A., Mahumane B., Kihamia C., Mwanri L., Tatala S., Lwambo N., Siza J., Khanh L.N., Khoi H.H., Toan N.D. (2001). Anaemia in schoolchildren in eight countries in Africa and Asia. Public Health Nutr..

[bib0115] Kapito-Tembo A.P., Mwapasa V., Meshnick S.R., Samanyika Y., Banda D., Bowie C., Radke S. (2009). Prevalence distribution and risk factors for Schistosoma hematobium infection among school children in Blantyre, Malawi. PLoS Negl. Trop. Dis..

[bib0120] Katz N., Chaves A., Pellegrino J. (1972). A simple device for quantitative stool thick-smear technique in Schistosomiasis mansoni. Revista do Instituto de Medicina Tropical de Sao Paulo.

[bib0125] Keiser J., N’Goran E.K., Traore M., Lohourignon K.L., Singer B.H., Lengeler C., Tanner M., Utzinger J. (2002). Polyparasitism with Schistosoma mansoni, geohelminths, and intestinal protozoa in rural Cote d’Ivoire. J. Parasitol..

[bib0130] King C.H., Bertsch D. (2013). Meta-analysis of urine heme dipstick diagnosis of schistosoma haematobium infection, including low-prevalence and previously-treated populations. PLoS Negl.Trop. Dis..

[bib0135] King C.H., Dangerfield-Cha M. (2008). The unacknowledged impact of chronic schistosomiasis. Chronic Illness.

[bib0140] King K.C., Stelkens R.B., Webster J.P., Smith D.F., Brockhurst M.A. (2015). Hybridization in parasites: consequences for adaptive evolution, pathogenesis, and public health in a changing world. PLoS Pathog..

[bib0145] Knopp S., Corstjens P.L., Koukounari A., Cercamondi C.I., Ame S.M., Ali S.M., de Dood C.J., Mohammed K.A., Utzinger J., Rollinson D., van Dam G.J. (2015). Sensitivity and specificity of a urine circulating anodic antigen test for the diagnosis of schistosoma haematobium in low endemic settings. PLoS Negl. Trop. Dis..

[bib0150] Koukounari A., Donnelly C.A., Sacko M., Keita A.D., Landoure A., Dembele R., Bosque-Oliva E., Gabrielli A.F., Gouvras A., Traore M., Fenwick A., Webster J.P. (2010). The impact of single versus mixed schistosome species infections on liver, spleen and bladder morbidity within Malian children pre- and post-praziquantel treatment. BMC Infect. Dis..

[bib0155] Koukounari A., Estambale B.B., Njagi J.K., Cundill B., Ajanga A., Crudder C., Otido J., Jukes M.C., Clarke S.E., Brooker S. (2008). Relationships between anaemia and parasitic infections in Kenyan schoolchildren: a Bayesian hierarchical modelling approach. Int. J. Parasitol..

[bib0160] Koukounari A., Fenwick A., Whawell S., Kabatereine N.B., Kazibwe F., Tukahebwa E.M., Stothard J.R., Donnelly C.A., Webster J.P. (2006). Morbidity indicators of Schistosoma mansoni: relationship between infection and anemia in Ugandan schoolchildren before and after praziquantel and albendazole chemotherapy. Am. J. Trop. Med. Hyg..

[bib0165] Landoure A., Dembele R., Goita S., Kane M., Tuinsma M., Sacko M., Toubali E., French M.D., Keita A.D., Fenwick A., Traore M.S., Zhang Y. (2012). Significantly reduced intensity of infection but persistent prevalence of schistosomiasis in a highly endemic region in Mali after repeated treatment. PLoS Negl. Trop. Dis..

[bib0170] Leger E., Webster J.P. (2017). Hybridizations within the Genus Schistosoma: implications for evolution, epidemiology and control. Parasitology.

[bib0175] Lengeler C., Utzinger J., Tanner M. (2002). Screening for schistosomiasis with questionnaires. Trends Parasitol..

[bib0180] Lwambo N.J., Brooker S., Siza J.E., Bundy D.A., Guyatt H. (2000). Age patterns in stunting and anaemia in African schoolchildren: a cross-sectional study in Tanzania. Eur. J. Clin. Nutr..

[bib0185] Magalhaes R.J., Clements A.C. (2011). Mapping the risk of anaemia in preschool-age children: the contribution of malnutrition, malaria, and helminth infections in West Africa. PLoS Med..

[bib0190] McDonald M.M., Swagerty D., Wetzel L. (2006). Assessment of microscopic hematuria in adults. Am. Fam. Physician.

[bib0195] Midzi N., Mtapuri-Zinyowera S., Mapingure M.P., Sangweme D., Chirehwa M.T., Brouwer K.C., Mudzori J., Hlerema G., Mutapi F., Kumar N., Mduluza T. (2010). Consequences of polyparasitism on anaemia among primary school children in Zimbabwe. Acta Trop..

[bib0200] Mupfasoni D., Karibushi B., Koukounari A., Ruberanziza E., Kaberuka T., Kramer M.H., Mukabayire O., Kabera M., Nizeyimana V., Deville M.A., Ruxin J., Webster J.P., Fenwick A. (2009). Polyparasite helminth infections and their association to anaemia and undernutrition in Northern Rwanda. PLoS Negl. Trop. Dis..

[bib0205] Oliveira D., Ferreira F.S., Atouguia J., Fortes F., Guerra A., Centeno-Lima S. (2015). Infection by intestinal parasites, stunting and anemia in school-aged children from Southern Angola. PloS One.

[bib0210] Olsen A., Magnussen P., Ouma J.H., Andreassen J., Friis H. (1998). The contribution of hookworm and other parasitic infections to haemoglobin and iron status among children and adults in western Kenya. Trans. R. Soc. Trop. Med. Hyg..

[bib0215] Peters P.A., Warren K.S., Mahmoud A.A. (1976). Rapid, accurate quantification of schistosome eggs via nuclepore filters. J. Parasitol..

[bib0220] Pullan R., Brooker S. (2008). The health impact of polyparasitism in humans: are we under-estimating the burden of parasitic diseases?. Parasitology.

[bib0225] Pullan R.L., Gitonga C., Mwandawiro C., Snow R.W., Brooker S.J. (2013). Estimating the relative contribution of parasitic infections and nutrition for anaemia among school-aged children in Kenya: a subnational geostatistical analysis. BMJ Open.

[bib0230] Pullan R.L., Kabatereine N.B., Bukirwa H., Staedke S.G., Brooker S. (2011). Heterogeneities and consequences of Plasmodium species and hookworm coinfection: a population based study in Uganda. J. Infect. Dis..

[bib0235] Richter J., Domingues A.L., Barata C.H., Prata A.R., Lambertucci J.R. (2001). Report of the second satellite symposium on ultrasound in schistosomiasis. Mem. Inst. Oswaldo Cruz.

[bib0240] Richter J., Hatz C., Campagne G., Bergquist N., Jenkins J. (2000). Ultrasound in Schistosomiasis: A Practical Guide to the Standardized Use of Ultrasonograpghy for the Assessment of Schistosomiasis Related Morbidity.

[bib0245] Sacko M., Magnussen P., Keita A.D., Traore M.S., Landoure A., Doucoure A., Madsen H., Vennervald B.J. (2011). Impact of Schistosoma haematobium infection on urinary tract pathology, nutritional status and anaemia in school-aged children in two different endemic areas of the Niger River Basin. Mali. Acta Tropica.

[bib0250] Scott J.T., Diakhate M., Vereecken K., Fall A., Diop M., Ly A., De Clercq D., de Vlas S.J., Berkvens D., Kestens L., Gryseels B. (2003). Human water contacts patterns in Schistosoma mansoni epidemic foci in northern Senegal change according to age, sex and place of residence, but are not related to intensity of infection. Trop. Med. Int. Health: TM & IH.

[bib0255] Soares Magalhaes R.J., Clements A.C. (2011). Spatial heterogeneity of haemoglobin concentration in preschool-age children in sub-Saharan Africa. Bull. World Health Organ..

[bib0260] Sogoba N., Doumbia S., Vounatsou P., Bagayoko M.M., Dolo G., Traore S.F., Maiga H.M., Toure Y.T., Smith T. (2007). Malaria transmission dynamics in Niono, Mali: the effect of the irrigation systems. Acta Trop..

[bib0265] Southgate V.R., Rollinson D., Tchuem Tchuente L.A., Hagan P. (2005). Towards control of schistosomiasis in sub-Saharan Africa. J. Helminthol..

[bib0270] SPRING (2016). MALI - National Anaemia Profile.

[bib0275] Stata Corp (2011). Stata Statistical Software: Release 12. StataCorp LP, College Station, TX..

[bib0280] Stecher C.W., Sacko M., Madsen H., Wilson S., Wejse C., Keita A.D., Landoure A., Traore M.S., Kallestrup P., Petersen E., Vennervald B. (2017). Anemia and growth retardation associated with Schistosoma haematobium infection in Mali: a possible subtle impact of a neglected tropical disease. Trans. R. Soc. Trop. Med. Hyg..

[bib0285] Stoltzfus R.J., Chwaya H.M., Tielsch J.M., Schulze K.J., Albonico M., Savioli L. (1997). Epidemiology of iron deficiency anemia in Zanzibari schoolchildren: the importance of hookworms. Am. J. Clin. Nutr..

[bib0290] Sturrock R.F., Mahmoud A.A.F. (2001). The schistosomes and their intermediate hosts. Schistosomiasis.

[bib0295] Teesdale C.H., Fahringer K., Chitsulo L. (1985). Egg count variability and sensitivity of a thin smear technique for the diagnosis of Schistosoma mansoni. Trans. R. Soc. Trop. Med. Hyg..

[bib0300] Thoradeniya T., Wickremasinghe R., Ramanayake R., Atukorala S. (2006). Low folic acid status and its association with anaemia in urban adolescent girls and women of childbearing age in Sri Lanka. Br. J. Nutr..

[bib0305] Traore M., Traore H.A., Kardorff R., Diarra A., Landoure A., Vester U., Doehring E., Bradley D.J. (1998). The public health significance of urinary schistosomiasis as a cause of morbidity in two districts in Mali. Am. J. Trop. Med. Hyg..

[bib0310] Tukahebwa E.M., Magnussen P., Madsen H., Kabatereine N.B., Nuwaha F., Wilson S., Vennervald B.J. (2013). A very high infection intensity of Schistosoma mansoni in a Ugandan Lake Victoria Fishing Community is required for association with highly prevalent organ related morbidity. PLoS Negl. Trop. Dis..

[bib0315] Ullah I., Zahid M., Sthanadar A.A., Sthanadar I.A., Ali P.A., Mudassirshah Khan M.I., Aslam M., Ullah W. (2014). Iron deficiency anemia in school age children in District Karak Khyber Pakh- tunkhwa Province, Pakistan. Open J. Blood Dis..

[bib0320] van der Werf M.J., de Vlas S.J., Brooker S., Looman C.W., Nagelkerke N.J., Habbema J.D., Engels D. (2003). Quantification of clinical morbidity associated with schistosome infection in sub-Saharan Africa. Acta Trop..

[bib0325] van der Werf M.J., de Vlas S.J., Landoure A., Bosompem K.M., Habbema J.D. (2004). Measuring schistosomiasis case management of the health services in Ghana and Mali. Trop. Med. Int. health: TM & IH.

[bib0330] Vandekerckhove J., Matzke D., Wagenmakers E., Busemeyer J.R., Townsend Z., Wang J., Eidels A. (2014). Model comparison and the principle of parsimony. Oxford Handbook of Computational and Mathematical Psychology..

[bib0335] Vennervald B.J., Dunne D.W. (2004). Morbidity in schistosomiasis: an update. Curr. Opin. Infect. Dis..

[bib0340] Verma M., Chhatwal J., Kaur G. (1998). Prevalence of anemia among urban school children of Punjab. Indian Pediatr..

[bib0345] von Schenck H., Falkensson M., Lundberg B. (1986). Evaluation of "HemoCue," a new device for determining hemoglobin. Clin. Chem..

[bib0350] WHO (2000). Ultrasound in Schistosomiasis: Second International Workshop, October 22–26, 1996, Niamy, Niger, A Practical Guide to the Standardised Use of Ultrasonography for the Assessment of Schistosomiasis-Related Morbidity.

[bib0355] WHO (2001). Iron Deficiency Anaemia: Assessment, Prevention and Control. Guidelines for Programme Managers.

[bib0360] WHO (2006). Preventive Chemotherapy in Human Helminthiasis. Coordinated Use of Anthelminthic Drugs in Control Interventions: A Manual for Health Professionals and Programme Managers.

[bib0365] WHO (2011). Haemoglobin Concentrations for the Diagnosis of Anaemia and Assessment of Severity. Vitamin and Mineral Nutrition Information System.

[bib0370] WHO (2012). Helminth Control in School-Age Children.

