# Supplementary material for: The association between child Schistosoma spp. infections and morbidity in an irrigated rice region in Mali: A localized study
Source: Acta Trop. 2019 Nov;199:105115. doi: 10.1016/j.actatropica.2019.105115 (PMC6995995; doi:10.1016/j.actatropica.2019.105115)
Supplement: Supplementary file 1 [file mmc1.docx]

**Supplement material**

**S.1** Akaike’s Information Criterion from the models fitting sex, age group and

infection intensity classes

|  | **Intensities of schistosome species as linear effects with interactions** | **Intensities of schistosome species as linear effects without interactions** | **Intensities of schistosome species as categorical effects** |
| --- | --- | --- | --- |
| ***Response variable*** | ***AIC (BIC)*** | ***AIC (BIC)*** | ***AIC (BIC)*** |
| Bladder pathology as assessed by positive global scores | 187.3* (206.9) | 185.4 (201.7) | 190.9 (217.1) |
| Anaemia as assessed by haemoglobin levels | 90.6° (110.1) | 93.6 (109.8) | 91.5 (117.4) |

*In all these models the interaction term was not significant.

**°**In this model the interaction term was significant.

BIC: Bayesian information criterion

**S.2** Multivariate logistic regression of anaemia pathology as assessed by HemoCue^®^ (n = 189)

| **Estimates from multilevel logistic regressions** | | | | |
| --- | --- | --- | --- | --- |
|  | **Adjusted OR for gender and age (95 % CI) and p-values for the risk of having anaemia pathology as assessed by haemoglobin levels** | | | |
| ***Type of intensity of schistosomiasis infection related variables*** | ***For S. mansoni*** | | | |
| ***For S. haematobium*** | none | light | medium | heavy |
| None | 1 | 2.045  (1.072 to 3.900)  p=0.030 | 4.183  (1.149 to 15.216)  p=0.030 | 8.555  (1.233 to 59.357)  p=0.030 |
| Light | 0.554  (0.213 to 1.442)  p=0.227 | 1.134  (0.413 to 3.108)  p=0.807 | 2.319  (0.573 to 9.381)  p =0.238 | 4.744  (0.689 to 32.662)  p=0.001 |
| Heavy | 0.307  (0.045 to 2.081)  p=0.227 | 0.628  (0.098 to 4.024)  p=0.624 | 1.286  (0.171 to 9.662)  p=0.807 | 2.630  (0.250 to 27.578)  p=0.420 |

ORs, Odds Ratios; 95% CIs, 95% Confidence Intervals

**S3 Multivariate logistic regression of bladder pathology as assessed by US (n = 194)**

| **Estimates from multilevel logistic regressions** | | | | |
| --- | --- | --- | --- | --- |
|  | **Adjusted OR for gender and age (95 % CI) and p-values for the risk of having bladder pathology as assessed by positive global scores** | | | |
| ***Type of intensity of schistosomiasis infection related variables*** | ***For S. mansoni*** | | | |
| ***For S. haematobium*** | none | light | medium | heavy |
| None | 1 | 0.828  (0.551 to 1.245)  p=0.366 | 0.687  (0.304 to 1.550)  p=0.366 | 0.569  (0.1681 to 1.930)  p= 0.366 |
| Light | 2.395  (1.264 to 4.536)  p=0.007 | 1.985  (1.021to 3.859)  p=0.043 | 1.645  (0.670 to 4.041)  p=0.277 | 1.364  (0.400 to 4.648)  p=0.001 |
| Heavy | 5.736  (1.599 to 20.579)  p=0.007 | 4.755  (1.378 to 16.411)  p=0.014 | 3.942  (1.043 to 14.898)  p=0.043 | 3.268  (0.709 to 15.047)  p=0.129 |

ORs, Odds Ratios; 95% CIs, 95% Confidence Intervals
